# Circadian Rhythm Dysregulation and Leukemia Development: The Role of Clock Genes as Promising Biomarkers

**DOI:** 10.3390/ijms23158212

**Published:** 2022-07-26

**Authors:** Ana Beatriz Aguiar Sanford, Leidivan Sousa da Cunha, Caio Bezerra Machado, Flávia Melo Cunha de Pinho Pessoa, Abigail Nayara dos Santos Silva, Rodrigo Monteiro Ribeiro, Fabiano Cordeiro Moreira, Manoel Odorico de Moraes Filho, Maria Elisabete Amaral de Moraes, Lucas Eduardo Botelho de Souza, André Salim Khayat, Caroline Aquino Moreira-Nunes

**Affiliations:** 1 Unichristus University Center, Faculty of Biomedicine, Fortaleza 60430-275, CE, Brazil; anabeatrizsanford@gmail.com (A.B.A.S.); leidivansc@gmail.com (L.S.d.C.); 2 Pharmacogenetics Laboratory, Department of Medicine, Drug Research and Development Center (NPDM), Federal University of Ceará, Fortaleza 60430-275, CE, Brazil; caio.bmachado97@gmail.com (C.B.M.); flaviamelop@outlook.com (F.M.C.d.P.P.); odorico@ufc.br (M.O.d.M.F.); betemora@ufc.br (M.E.A.d.M.); 3 Department of Biological Sciences, Oncology Research Center, Federal University of Pará, Belém 66073-005, PA, Brazil; ssnayara23@gmail.com (A.N.d.S.S.); fabiano.ufpa@gmail.com (F.C.M.); khayatas@gmail.com (A.S.K.); 4 Department of Hematology, Fortaleza General Hospital (HGF), Fortaleza 60150-160, CE, Brazil; rmonteiroribeiro@icloud.com; 5 Center for Cell-Based Therapy, Regional Blood Center of Ribeirão Preto, University of São Paulo, São Paulo 14051-140, SP, Brazil; lucas.souza@hemocentro.fmrp.usp.br; 6 Northeast Biotechnology Network (RENORBIO), Itaperi Campus, Ceará State University, Fortaleza 60740-903, CE, Brazil

**Keywords:** circadian rhythm, leukemia, clock genes, cell cycle, biomarkers

## Abstract

The circadian clock (CC) is a daily system that regulates the oscillations of physiological processes and can respond to the external environment in order to maintain internal homeostasis. For the functioning of the CC, the clock genes (CG) act in different metabolic pathways through the clock-controlled genes (CCG), providing cellular regulation. The CC’s interruption can result in the development of different diseases, such as neurodegenerative and metabolic disorders, as well as cancer. Leukemias correspond to a group of malignancies of the blood and bone marrow that occur when alterations in normal cellular regulatory processes cause the uncontrolled proliferation of hematopoietic stem cells. This review aimed to associate a deregulated CC with the manifestation of leukemia, looking for possible pathways involving CG and their possible role as leukemic biomarkers.

## 1. Introduction

The circadian rhythm, or circadian clock (CC), is a hierarchically cyclic system that regulates the daily oscillations of physiological processes and can respond to external environment changes to maintain internal homeostasis [1]. Because some of these outside shifts are caused by the 24-h rotation of the Earth, and so are expected to occur approximately at the same time every day, the circadian regulation allows the human organism to anticipate these changes and synchronize them with inner physiology [2].

Starting in the 1980s, studies that culminated in the characterization of the first clock gene (CG) in *Drosophila melanogaster* paved the way for the characterization of additional genes and proteins, leading to what we now know as the circadian clock [3,4,5].

For the control, the activation and repression of CC genes may need environmental stimuli—known as zeitgebers (ZTGBs)—which can be a photic or a non-photic stimulus, where the most studied are the photic stimuli, or the light/dark cycle [6,7]. They are accountable for sending signals to the suprachiasmatic nucleus (SCN) in the central nervous system, stimulating CG transcription located in peripheral tissues, and promoting cell metabolic functions [8].

The CC response to ZTGBs depends on the strength of the stimulus and the circadian phase during which it is applied. In addition to being able to synchronize the circadian rhythm under normal conditions, these principles form an adaptive advantage that the CC transmits to the body. However, erroneous exposure to ZTGBs can disturb circadian homeostasis and have harmful effects on human health [9].

Furthermore, the disruption of the CC in mammals can result in the development of different diseases, such as neurodegenerative and metabolic disorders, as well as cancer. There is evidence that artificial light, shift work, and jet travel can contribute to circadian rhythm misalignment [10,11].

## 2. Circadian Clock Genes

The CC can be divided into two operation levels: systemic and cellular [12]. The central clock at a systemic scale, known as the “master” clock, is regulated by the SCN in the anterior hypothalamus [12,13,14], and is responsible for coordinating the cell-autonomous clocks in peripheral tissues, as well as other brain areas, directly through neural and humoral signs, when it is given a stimulus from an external environment, such as light change, temperature, sleep deprivation, and feeding [6,9,15].

At the cellular level, the CC is regulated by positive and negative transcription-translation feedback loops (TTFL), which control the rhythmicity of cellular, metabolic, and physiologic events [13,14,16]. The clock is driven positively by the transcription of the basic helix-loop-helix (bHLH) genes *CLOCK* (circadian clock regulator) and *BMAL1*/*ARNTL* (brain and muscle Arnt-like protein-1, also known as aryl hydrocarbon nuclear receptor translocator-like), and the Per-Arnt-Sim (PAS) gene *NPAS2* (neuronal PAS domain protein 2) [17,18,19,20,21,22,23]. *CLOCK/BMAL1* and *NPAS2/BMAL1* form a heterodimer that, when occupying the Enhancer box (E-box) regions on their promoting target, will stimulate the expression of the core clock genes, such as *PER1/2/3* (period circadian clocks 1, 2, and 3, respectively), *CRY1*/*2* (cryptochrome circadian clocks 1 and 2, respectively), and *TIM* (timeless) [24,25,26,27,28,29,30]. The higher expression of *PER*, *CRY*, and *TIM* allows them to bind to the CLOCK-BMAL1 and NPAS2-BMAL1 complexes and inhibit their expression, constituting the negative control [31,32,33].

**Figure 1:**
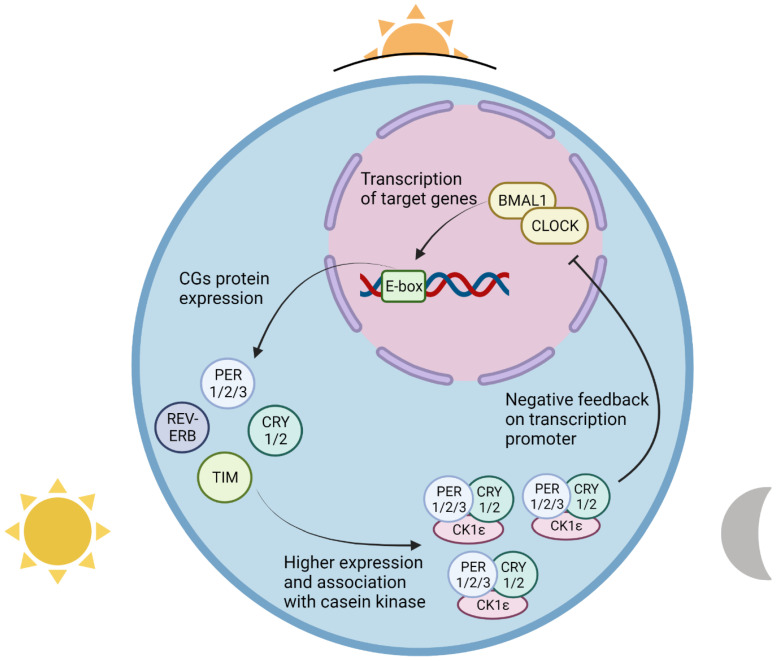
Clock-BMAL1 complex activity during a day cycle. At sunrise, CLOCK-BMAL1 promotes the beginning of the circadian clock by binding to the enhancer box (E-box) regions of target circadian clock genes (CG) and increasing their transcription. During the day, the cellular levels of clock proteins such as PER 1/2/3, CRY 1/2, TIM, and REV-ERB continue to rise; at the day’s end, their association with casein kinase 1 epsilon (CK1ε) facilitates transportation into the cell nucleus, where circadian clock gene proteins have a negative feedback effect on the activity of the CLOCK-BMAL1 complex. The suppressive effect of the PER-CRY-CK1ε complex on transcription factors leads to their inhibition of their own transcription, and a consequent decrease in the complex’s cell levels during the night and until dawn, after which the activity of CLOCK-BMAL1 is once again unimpaired, and the circadian clock restarts. Created with BioRender.com.

In the early hours of dawn, even with the binding of the CLOCK-BMAL1 complex in its target E-box sequence, the high levels of CRY and PER bind to this complex and inhibit their transcription, creating the repressive regulation [34]. Consequently, CRY and PER repress their own expression, and by the sunrise, the lack of production of CRY and PER proteins causes reduced levels in the cell nucleus and, in the absence of binding to CLOCK-BMAL1, allows these complexes to start their transcription, creating the active regulation [8,34]. In the early evening, due to the high expression of CG throughout the day, the levels of CRY and PER rise again; in this way, they manage to enter the nucleus by binding with casein kinase 1 epsilon (CK1ε), constituting the PER-CRY-CK1ε complex, and they suppress the positive transcription of CLOCK-BMAL1, thus restarting another cycle (Figure 1) [35].

This daily loop is also regulated by *REV-ERBα/β* (nuclear receptor subfamily 1 group D members 1 and 2, also known as NR1D1 and NR1D2, respectively), and *RORα/β/γ* (retinoic acid-related orphan nuclear receptors alpha, beta and gamma) [31,36,37,38,39,40]. *REV-ERBs* are responsible for *BMAL1* transcriptional repression, through the recruitment of nuclear receptor corepressor (NCoR) and histone deacetylase 3 (HDAC3), while *RORα* stimulates *BMAL1* expression, competing with *REV-ERBs* [11,41,42]. Furthermore, there are more genes that also play a role in the circadian clock [7]. *SHARP1*, also known as *DEC2* or *BHLHE41*, is a bHLH transcription factor expressed rhythmically in SCN and peripheral tissues, which is included in the clock genes’ negative control, where it can bind to BMAL1 or to the E-box present in the CLOCK/BMAL1 and NPAS2/BMAL1 heterodimers, inhibiting its expression and, consequently, *PER* and *CRY* expression [43,44,45,46,47].

Another example is peroxisome proliferator-activated receptors (PPARs), also known as NR1Cs, which are nuclear receptors that, when activated by their respective ligands, have a role in the regulation of gene expression in the cell nucleus [48,49]. *PPAR* has three different isoforms in mammals—PPARα (NR1C1), PPARβ (NR1C2), and PPARγ (NR1C3)—which are expressed at different levels in tissues and have different roles in the circadian rhythm, especially in *BMAL1* and *REV-ERBs* expression [48,49,50,51,52]. These mechanisms help to control the activation/inhibition of the CGs, providing homeostatic maintenance in mammals [53,54].

### 2.1. Clock Gene Dysregulation and Disease Development

The CC controlled by CGs has evolved over hundreds of millions of years by efficiently orchestrating the metabolism, separating and dividing metabolic processes like anabolism and catabolism, and optimizing the metabolic efficiency of periods of feeding and fasting [55].

Thus, the CC comprises a central pacemaker in various tissues and modulates a wide range of metabolic targets, including insulin sensitivity, cholesterol synthesis, fat oxidation, and energy expenditure. Furthermore, there is evidence to suggest that the disruption of the CC increases the risk of metabolic diseases, proving once again that it is an important physiological regulator [55].

The CC can also cause the misalignment of nutrient flows and contribute to the pathophysiology of type 2 diabetes mellitus at the tissue level. Insulin resistance is also an important determinant of the disease. CLOCK and BMAL1 regulate the muscle’s sensitivity to insulin through changes in protein levels and membrane translocation of the insulin-sensitive glucose transporter (GLUT4). There is also an influence of the CC on the interruption of the pancreatic clock, causing a defect in insulin secretion [26,53,56].

Studies have shown that the interaction of CGs and neoplasms—including many cellular functions, such as the cell cycle and cell division—are controlled in part by components of the molecular clock [26,56,57]. In the cell cycle, CGs act as critical regulators in different phases, where they can either stimulate or stop cell proliferation [58]. The cell cycle consists of four main phases—G1 (pre-replicative), S (synthesis), G2 (post-replicative), and M (mitosis and cell division)—mainly controlled by the activity of cyclins, cyclin-dependent kinases (CDKs), and CDK inhibitors (CDIs) [59,60]. In between each of these phases there are checkpoints that evaluate whether the DNA replication was successful and decide whether the cell will continue to the next phase or if the cycle will be interrupted [61,62]. These cell cycle control mechanisms have been shown to have genes that can be regulated by CGs, called clock-controlled genes (CCGs) [63]. The CCGs, specifically *Myc*, *Wee1*, *p21*, *p53*, and *cyclins* genes, have already been reported in other types of cancer, in which their expression or suppression can contribute to pathology development and progression [63,64,65,66]. Thus, uncontrolled cell proliferation is a hallmark of cancer, and the rate of proliferation depends on the cell cycle [67,68,69].

It is worth mentioning that the CC is closely linked to physiology, such as in liver function, which follows a daily rhythm. Changes in the liver clock result in metabolic disorders such as non-alcoholic fatty liver disease and impaired glucose metabolism, which can cause the activation of oncogenic pathways, inducing spontaneous hepatocarcinoma [8,70]. The hypothesis is that this interruption—through the suppression of melatonin—modulates sex hormones and changes the expression of peripheral cells and of all cellular physiology mediated by CGs [10,71].

Other studies have also shown that shift work and changes in CGs can be described as risk factors for the development of cancer, such as skin cancer-like squamous cell carcinoma, melanoma, basal cell carcinoma [72], and gastric cancer [73]. Studies have also reported an influence of the CC on colorectal cancer, showing that proteins such as CLOCK1, BMAL1, PER, and CRY have various effects on the *c-Myc/p21* and Wnt/β-catenin pathways and influence several steps of the DNA damage response, thus playing a critical role in the preservation of genomic integrity [74,75].

Another point to be highlighted is the studies that demonstrate the influence of CGs on cell proliferation and migration in lymphatic cancers, such as Hodgkin’s lymphoma and non-Hodgkin’s lymphoma [76,77], as well as studies that established a role for the central genes of the CC in leukemias [76,78].

Therefore, some components of the CC have a significant antitumor effect through cell cycle arrest, the DNA damage response, and correlation with important pathways, including a PER and p53 relation [75,79,80], and data like this support a possible role of circadian CG in the development and critical aspects of several types of cancer, including leukemias.

Even with evidence that neoplastic formation is correlated with changes in the CC, there are few therapeutic approaches that use this correlation; thus, it is emerging as a possible promising approach for the treatment of cancer, in which anticancer drugs are administered at the ideal time according to the CC; this is called ‘chronotherapy’, and it can be especially beneficial when we associate tolerance to chemotherapy and a more adequate action. Therefore, while the inclusion of chronotherapy in cancer therapy may offer a more effective and less toxic approach, biomarkers for chronotherapy strategies’ efficiency still need to be properly defined [81,82,83,84].

### 2.2. Clock Genes’ Role in the Leukemia Pathway

Leukemias are a group of malignancies of the blood and bone marrow that occur when alterations in normal cellular regulatory processes cause the uncontrolled proliferation of hematopoietic stem cells. This group of diseases is usually classified into subtypes defined by cell lineage that can be either lymphocytic or myeloid, and according to the type and stage of cell maturation, with these being acute or chronic [85,86].

Among the causes, leukemias might be developed because of reciprocal chromosomal errors or chromosomal deletions, in addition to point mutations and epigenetic alterations. It is often assumed that these errors in cancer cells are caused by uncontrolled cell cycle progression, mainly because cell cycle checkpoints fail and lead the cell to become cancerous [60,85,86,87].

We already know that the CG and the cell cycle are robustly coupled, cooperating for proper cell functioning, and the dysregulation of the CC can affect cell homeostasis and promote cancer development [88]. Therefore, even though the functions of the CC in normal physiology are clear, studies that portray its alterations in cancer, including leukemias, are lacking, leaving a gap in the clarity and description of their functions in neoplastic cells. For this reason, the aim of this study is to investigate and identify the role of circadian rhythm clock genes in leukemia development, and the further implications of this.

## 3. Results

In order to investigate the possible role of CGs in leukemia development, a search was carried out in the PubMed database with the keywords “clock genes”, “circadian rhythm” and “leukemia”, which initially found 36 articles from the last 10 years. The inclusion criteria were clinical and preclinical studies that used leukemia cell lines or samples from patients with leukemia; therefore, the number of results was reduced to eight studies, all of which are included in Table 1.

The resulting data portray alterations in the CGs in four types of leukemias: acute myeloid leukemia (AML), chronic myeloid leukemia (CML), acute lymphocytic leukemia (ALL), and chronic lymphocytic leukemia (CLL). The samples collected were cell lines, peripheral blood, and bone marrow from leukemia patients and control groups (healthy subjects). The CGs *BMAL1*, *PER1*, *PER2*, *PER3*, *NAPS2*, *TIM*, *CRY1*, *CRY2*, *CKIε*, *CLOCK*, *REV-Erα*, *PPARα*, and *SHARP1* were the more frequent genes described in the pointed studies; their function was correlated with their expression levels in leukemia patients.

## 4. Discussion

### 4.1. Role of PER Genes in Leukemia

The *Period* genes, as described before, are important to control the negative expression of the circadian clock, and disruption in these genes’ expression will lead to dysregulated clock-controlled gene expression [32,97]. These alterations can favor cancerous cells’ proliferation and life, whereas the defective cells will not undergo apoptosis [59,98]. For instance, PER1 and PER2 proteins have already been described as tumor suppressors in cancer cell cultures, as well in animal experiments [99,100,101,102].

In the cell cycle, high levels of PERs inhibit *c-Myc* gene transcription by binding to its E-box sequence, stopping *cyclin D1* expression and cell proliferation and, when necessary, inducing apoptosis [103,104]. On the contrary, when the levels of PERs are low, the *c-Myc*/*cyclin D1* mechanism will be overactivated, which will increase cell proliferation [98,105].

In the studies found, PERs were shown to be significantly downregulated in all four types of leukemia patients compared to healthy controls [89,91,92,96]. In addition, Rana et al. [89] also related an up-regulation of *Myc* and *cyclin D1* expression in CLL patients compared to the controls. These results agree with the pathophysiology of the PER genes already described in other types of cancer, where decreased PER expression seems to be related to increased aggressiveness and a worse prognosis, indicating that the same mechanism probably also occurs in leukemia [6,39,74,106]. Accordingly, Yang et al. [91] demonstrated that patients afflicted with either AML or ALL only had elevated levels of PER1 and PER3 at the end of their treatment, respectively, indicating that this high expression of the *Period* genes could also play an active role in tumor suppression in leukemia, as described in other cancer types [99,100,101]. In this way, the *PER* genes could become a possible biomarker for the suggestion of the pathology development when the PER levels are low, as well for a positive response in leukemia therapy when their levels are increased.

### 4.2. Cryptochrome Genes’ Expression in Leukemia

Unlike the *PER* genes, *Cryptochrome* genes presented divergent results. Although *CRY1* and *CRY2* were down-regulated in most of the identified results in leukemia patients, some cases showed a high expression of these genes [91,92,93]. Firstly, Habashy et al. [93] reported different levels of CRY1 expression among CLL patients: 54 showed down-regulation and 40 showed up-regulation. Secondly, Rahman et al. [92] related an up-regulation of CRY2 in patients with the relapse of AML in patients with 3 months of chemotherapy for CML. It is important to keep in mind that *CRY1* and *CRY2* have distinct functions outside the circadian clock; as such, they need to be evaluated separately [107].

For instance, *CRY1* has been recently shown to be an important paper in cell cycle regulation, through DNA-damage response [108]. Its depletion reduced *p21* expression in osteosarcoma cells, which led to increased cell proliferation, suggesting its role as a tumor suppressor [109]. On the other hand, high levels of *CRY1* expression were found in gastric and cervical cancer, and were associated with a poor prognosis [73,110]. Furthermore, it is possible that the deregulation of *CRY1* expression, either high or low, could lead to leukemia progression through different mechanisms, but more studies are required to verify the action of this gene in the cell cycle.

In addition, the regulation of inflammation by the immune system in association with CC desynchrony has been proposed. The assessment of the levels of pro-inflammatory cytokines such as IL-1β, GM-CSF, IL-12, and IL-13 was high in a group of animals exposed to CC interruption, in contrast with the low levels in anti-inflammatory cytokines such as IL -10 [111]. Furthermore, abnormalities of cytokine and growth factor signaling pathways are characteristic of all forms of leukemia: lymphoid and myeloid, acute and chronic, where in normal hematopoietic cells, cytokines provide the stimulus for proliferation, survival, auto-renewal, differentiation, and activation [112].

It is worth mentioning that the impaired expression of CRY1 was associated with elevated levels of pro-inflammatory cytokines, such as tumor necrosis factor alpha (TNF-α), which can mediate pro-survival or pro-death signals after tumor target recognition. In addition to being produced by a wide variety of malignant cells and immune cells within the tumor-associated microenvironment, TNF-α can be produced by leukemia cells. Polymorphisms in the TNF-α promoter have been described in leukemia, as the frequency of TNF-α-308 G/A polymorphism in ALL and CLL cases is associated with a higher risk of death. In addition, in several clinical observations, the TNF-α expression levels positively correlated with adverse clinical endpoints in leukemia [113,114,115,116].

Interleukin-6 (IL-6), which plays a role in chronic inflammation, is closely related to cancer. In leukemias, it plays an important role in the network of cytokines involved in the regulation of hematopoiesis and leukemic blast formation [83,115]. It is noteworthy that aberrant cytokine signaling aids in chemotherapy resistance [112]. Thus, understanding these signaling pathways is a prerequisite for the development and rational use of molecularly targeted therapies in leukemia.

As for *CRY2*, its impairment in mouse fibroblast carries into an increased expression of *c-Myc*, resulting in greater cell growth, proponing its contribution to tumor limitation through *c-Myc* turnover [117]. Besides this, the down-regulation of *CRY2* was present in breast cancer and osteosarcoma [118,119]. However, *CRY2* up-regulation is described in chemoresistant colorectal cancer patients [120]. In addition, Chan et al. [121] showed that mutations in CRY2 led to *p53* suppression and provided cell proliferation. Thus, the different expression levels of *CRY1* and *CRY2* appear to impact leukemic cells over different pathways, showing the need for further investigation into these mechanisms in order to elucidate their value as a leukemic biomarker [122].

### 4.3. BMAL1, Clock, and NPAS2

As for *BMAL1* and *CLOCK* genes, both were shown to be downregulated in all types of leukemia [89,91,92]. These bHLHs have already been described in the literature as being able to play a role in cell cycle control [16,59,78]. The up-regulation of BMAL1/CLOCK activates the expression of the clock-controlled gene *Wee1*, binding with its E-box sequence [98,123]. In this way, the resulting proteins will phosphorylate CDK1/cyclin B and, consequently, inhibit the transition from the G2 phase to the M phase in the cell cycle, interrupting it [35,124]. Because of the low expression of *BMAL1* and *CLOCK* in the results found, it is possible that this deregulated mechanism aids in the continuous cell proliferation of leukemic cells, as it has been shown in other types of cancer [125,126,127]. Contributing to this hypothesis, Rana et al. [89] also showed reduced levels of *Wee1* expression in CLL patients compared to controls. Therefore, the down-regulation of *CLOCK* and *BMAL1* could be used as a biomarker in leukemia to support the identification of the disease; thus, the elevation of its expression compared to normal levels can be used to follow the response to treatments.

*NPAS2* is a functional analog of *CLOCK,* and it participates in the expression of clock genes [18], as we stated before. Furthermore, *NPAS2* also has a paper on the DNA damage response in cell cycle control [128,129]. The BMAL1/NPAS2 heterodimer, like *PERs* genes, can suppress *c-Myc* expression by binding with their E-box, causing cell cycle arrest [130,131]. The dysregulation of the BMAL1/NPAS2 heterodimer is associated with high levels of the *c-Myc* gene in cells, leading to genomic instability and an increase in tumorigenesis [131,132,133,134]. Thus, the overexpression of NPAS2 is present in different types of cancer, indicating that the same mechanism could also occur in leukemia [135,136,137,138].

Song et al. [95] showed that *NPAS2* was up-regulated in AML patients compared to controls, and that *NPAS2* knockdown in AML cells led to cell cycle arrest at the G1 and G2 phases, as well as apoptosis in leukemic cells, which may suggest the participation of this gene in the development of leukemia, demonstrating its possible role as biomarker of the disease. Moreover, the study also investigated the role of *NPAS2* in the cell division cycle 25A (CDC25A), a dual-specificity phosphatase that participates in the progression of the G1 to the S phase together with CDKs, and which may have its expression regulated by transcription factors [139,140,141,142]. High levels of CDC25A have already been reported in tumorigenic cells [143,144,145,146,147]. The study also related a downregulation of *CDC25A* expression in NPAS2-knockdown AML cells, which leads to proliferation suppression and cell death, suggesting that NPAS2 has an effect on CDC25A transcription.

In addition, some studies have already demonstrated different *NPAS2* polymorphisms’ expression as a risk biomarker in other types of cancer [148,149,150,151,152,153]. In the studies we found, Rana et al. [89] didn’t observe any significant difference in rs2305160 polymorphism in CLL patients compared to healthy individuals, and therefore couldn’t associate it with the risk of CLL. However, the authors pointed out that this difference may be due to the variance of the population in their study compared to previous searches, as well as a relatively small number of samples (*n* = 74); further investigations are required in order to elucidate the divergence and prove the usefulness of *NPAS2* polymorphisms as risk biomarkers [89].

### 4.4. REV-ERBα and PPARα

In our findings, only one study reported the expression level of *REV-ERBα* and *PPARα* [92]. In addition to the previously described role of REV-ERBs as being part of the negative control of *BMAL1*, they have also been characterized as being able to control *p21* expression [75]. This gene produces a protein that, when activated, inhibits the transition from the G1 to the S phase, causing cell cycle arrest [154]. REV-ERBs can inhibit p21 transcription and promote cell proliferation [155]. As for the *PPARα* genes, they can directly repress BMAL1 and REV-ERBα transcription, binding to the PPAR-responsive elements (PPRE) present in these clock genes [156,157,158]. Rahman et al. [92] showed the down-regulation of both clock genes, which suggests that *BMAL1* expression levels are not controlled by REV-ERBα and PPARα in leukemia, as is the case in some metabolic diseases, but by another mechanism that needs further investigation [51,159]. Thus, due to the limited data, the role of *REV-ERBα* and *PPAR*α as leukemia biomarkers is unclear, and requires more trials for its elucidation.

### 4.5. TIMELESS, CKIε, and SHARP1

The TIMELESS gene is described for its function in the regulation of the cell cycle, together with *PER* genes, specifically in the DNA damage response, where it is required, to the activation of Checkpoint kinase 1 (Chk1) and Checkpoint kinase 2 (Chk2), by binding with the serine/threonine-protein kinases *ATR* (ataxia telangiectasia and Rad3-related) and *ATM* (ataxia telangiectasia mutated), respectively [160,161]. Chk1 and Chk2, therefore, will be responsible for the cell cycle arrest in DNA damage, inactivating CDKs and providing time for the DNA repair [159].

Yang et al. [91] showed the different levels of *TIM* expression in two different types of leukemia; it was down-regulated in AML patients, but it was up-regulated in ALL patients, compared to healthy controls. This contrast could be due to the distinct pathophysiology of AML and ALL, where *TIM* could have different mechanisms in each subtype. For instance, a high expression of *TIM* has already been found in lung, cervical, and ovarian cancer cells, and is associated with its worse prognosis [160,162,163,164]. The upregulation of *TIM* could cause the greater inhibition of *CLOCK*, which would lead to a lack of *Wee1* expression and, consequently, allow the leukemic cells to constantly replicate due to impaired *Wee1* activity in the regulation of cell cycle checkpoints [165]. Besides this, the downregulation of TIM wouldn’t enable the Chk1 activation and, therefore, the cell would continuously proliferate, even with the DNA damage [166]. Thus, *TIM* silencing caused cell cycle arrest in the glioma cells’ G0 and G1 phases [167]. Either way, both hypotheses need further investigations to prove their accuracy before we will be able to assess their role as leukemia biomarkers.

*CKIε*, as said before, has an important paper in the circadian clock, alongside *PER* and *CRY* [35]. Furthermore, *CKIε* is also responsible for PERs’ phosphorylation in order to control the levels of the proteins in the cell [168,169,170]. Therefore, high levels of CKIε promote major PER degradation, and consequently increase the levels of cyclin D1 and cyclin E in cancer cells, promoting its proliferation [171]. In the findings, *CKIε* was upregulated in AML and ALL patients compared to healthy individuals; thereby, it is possible to have the same oncogenic function as reported in other cancerous malignancies, showing its potential function as a biomarker in leukemia [91].

As for *SHARP1*, Numata et al. [94] related up-regulation in MLL-AF6 AML, a type of acute leukemia where an abnormal chromosomal translocation is fused into the N-terminal portion of the mixed-lineage leukemia gene (*MLL*), adding a sequence that encodes the C-terminus of different fusion partner proteins: in this case, the AF6 protein gene [94,172]. As said earlier, SHARP1 can modulate the negative expression of the CLOCK/BMAL1 heterodimer [173,174]. Together with the previous results related to where *CLOCK* and *BMAL1* were down-regulated, it is possible that the up-regulation of *SHARP1* is also involved in this repression mechanism, suggesting its oncogenic role in leukemia and its role as a biomarker, although further research is needed to verify it [89,91,92,175,176,177].

**Figure 2:**
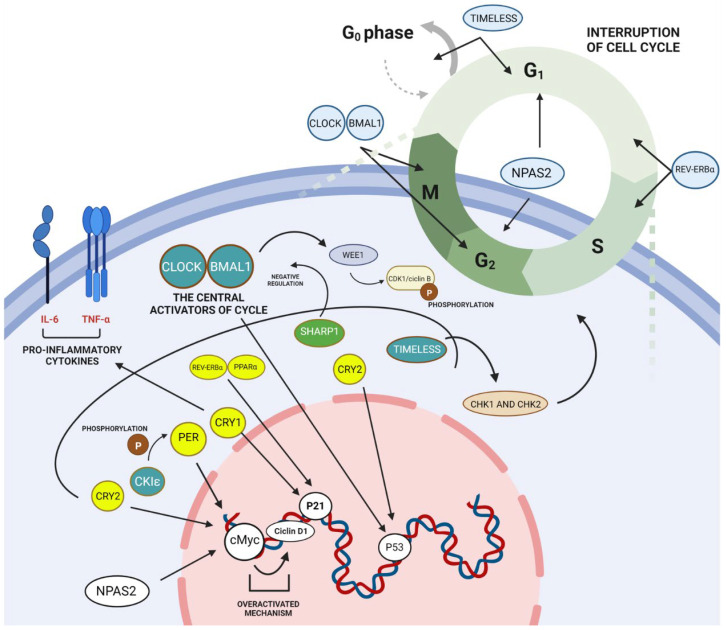
Role of clock genes in leukemic transformation. The CLOCK-BMAL1 complex promotes the expression of clock genes and changes in the circadian clock. Furthermore, at high expression levels, CLOCK-BMAL1 can directly affect Wee1, which phosphorylates CDK1/cyclin B, interfering at the cell cycle transition from the G2 to the M phase. In addition, some circadian clock genes were reported at low levels, represented in yellow as PER, which altered the expression of cMyc, which is closely linked to cyclin D1. This also happens with NAPS2, which affects cMyc-Cyclin D1, leaving it overactivated. Cry1 presents a low-level expression that directly affects the expression of p21 and pro-inflammatory cytokines such as TNF-α and IL-6. Also at low expression levels, Cry2 can affect cMyc expression, which again supports its overactivity. In addition, Cry2 also affects p53 expression. Low levels of REV-ERBα and PPARα can also be correlated with changes in p21 expression. Furthermore, the REV-ERBα gene can also inhibit the cell cycle transition from the G1 to the S phase. The *SHARP1* at high expression levels, as represented in green, has a correlation with the negative expression of CLOCK-BMAL1. The *TIMELESS* gene is also an important molecular biomarker, which—according to the studies—can present a high or low expression, participate in cell cycle checkpoints, and regulate Chk1 and Chk2, so it can cause an interruption in the G0 and G1 phases of the cell cycle. Finally, another gene that was shown to be at high and low levels was the CKIε gene, which—at its high levels—plays a role in the phosphorylation of the PER gene. Created with BioRender.com.

As described, leukemia is a hematological disease that occurs with the malignant transformation of white blood cells, where the molecular pathophysiology is not well established [85,86]. This review aimed to associate the dysregulated circadian rhythm with leukemia development, looking for possible molecular pathways involving the clock genes and their potential role as leukemic biomarkers. From these leukemia studies that described significant molecular changes in CGs’ expression, we were able to point out their possible molecular roles in the disease’s pathophysiology; Figure 2 represents some of the CGs’ molecular pathways and their regulation of cell cycle progression, as well as the possible induction of leukemogenesis.

## 5. Conclusions

In this review, clock genes have been proven to have an important influence on cell cycle progression, and consequent dysfunctions in CGs lead to changes in the normal cell functioning, often inducing constant cell proliferation and preventing apoptosis. *PER1/2/3*, *BMAL1*, *CLOCK*, *REV-ERBα*, and *PPARα* were downregulated in all types of leukemia, suggesting their potential role in leukemic development. Moreover, *NPAS2*, *CKIε*, and *SHARP1* were upregulated in leukemia, but their expression was limited to one or two subtypes of leukemia. In addition, *CRY1/2* and *TIM* genes showed different regulation levels in leukemic cells; thus, more investigation is essential in order to display and confirm their function in the disease. In short, this article pointed to the CGs as promising leukemia biomarkers; however, additional in vitro and in vivo experiments, as well as clinical trials, are required for a better elucidation of CGs’ involvement in leukemia biology, aiming to improve the diagnosis, prognosis, and patients’ clinical follow-up.

## Figures and Tables

**Table 1:** Studies on the involvement of clock genes in leukemia pathogenesis, from the past 10 years.

Gene	Type of Samples Analyzed	Function	Expression Levels	Reference
*BMAL1*	37 Blood samples from chronic lymphocytic leukemia. Patients and healthy controls	It forms a heterodimer with CLOCK and NPAS2. This heterodimer binds to E-box enhancing elements upstream of the Period (PER1, PER2, PER3) and Cryptochrome (CRY1, CRY2), activating the transcription of these genes. PER and CRY proteins heterodimerize and repress their own transcription by interacting in a feedback loop with CLOCK/BMAL1 or NPAS2/BMAL1 complexes.	Significantly downregulated in CLL patients as compared to their healthy controls. Not significantly altered in shift-workers as compared to non-shift-workers within the CLL group.	[89]
*PER1*		Encodes components of the circadian rhythms of locomotor activity, metabolism, and behavior.		
*PER2*				
*NPAS2* (rs2305160)	Blood sample of 74 individuals including 37 diagnosed cases of CLL	Probable involvement of NPAS2 in tumorigenesis, by regulating PER2 that can act as a tumor suppressor.	No significant association of rs2305160 polymorphism of NPAS2 gene with melatonin levels in any of the CLL groups.	[90]
*PER1*	Peripheral blood samples of 51 healthy adult volunteers, 44 patients newly-diagnosed with AML, and 23 newly–diagnosed with ALL	Encodes components of the circadian rhythms of locomotor activity, metabolism, and behavior.	Upregulated in patients with AM, who achieved remission, but remained low in patients whose disease relapsed after treatment. Downregulated in PB leukocytes in patients with AML when compared to those from healthy individuals.	[91]
*PER2*			Downregulated in PB leukocytes in patients with AML when compared to those from healthy individuals.	
*PER3*		Encodes components of the circadian rhythms of locomotor activity, metabolism, and behavior.	Upregulated in patients with ALL who achieved remission but remained low in patients whose disease relapsed after treatment. Downregulated in PB leukocytes in patients with AML when compared to those from healthy individuals. In patients with ALL, the expression levels were downregulated	
*TIM* (Timeless)		The protein encoded by this gene is highly conserved and is involved in cell survival after damage or stress, increase in DNA polymerase epsilon activity, maintenance of telomere length, and epithelial cell morphogenesis. The encoded protein also plays a role in the circadian rhythm autoregulatory loop, interacting with the PERIOD genes (PER1, PER2, and PER3) and others to downregulate activation of PER1 by CLOCK/BMAL1.	Downregulated in PB leukocytes in patients with AML when compared to those from healthy individuals. Upregulated in PB leukocytes in patients with ALL when compared to healthy individuals	
*CRY1*		This gene encodes a flavin adenine dinucleotide-binding protein that is a key component of the circadian core oscillator complex, which regulates the circadian clock	Downregulated in PB leukocytes in patients with AML when compared to those from healthy individuals. In patients with ALL, the expression levels were downregulated	
*CRY2*			Downregulated in PB leukocytes in patients with AML when compared to those from healthy individuals	
*BMAL1*		It forms a heterodimer with CLOCK and BMAL1. This heterodimer binds to E-box enhancing elements upstream of the Period (PER1, PER2, PER3) and Cryptochrome (CRY1, CRY2) genes and activates the transcription of these genes. PER and CRY proteins heterodimerize and repress their own transcription by interacting in a feedback loop with CLOCK/BMAL1 or NPAS2/BMAL1 complexes		
*CKIε*		Has been implicated in the control of cytoplasmic and nuclear processes, including DNA replication and repair. The encoded protein is found in the cytoplasm as a monomer and can phosphorylate a variety of proteins, including itself. This protein has been shown to phosphorylate the period (PER), a circadian rhythm protein.	Upregulated in PB leukocytes in patients with AML, when compared to those from healthy individuals. Upregulated in PB leukocytes in patients with ALL when Compared to healthy individuals	
*PER2*	Peripheral blood mononuclear cells (PBMCs) isolated from peripheral blood samples collected from 26 AML patients, 22 ALL patients, 13 CML patients, 14 CLL patients, and 30 healthy donors	Encodes components of the circadian rhythms of locomotor activity, metabolism, and behavior.	Downregulation in newly diagnosed patients with AML and patients with relapse of the disease, compared to controls. Downregulation in newly diagnosed and end of treatment from ALL patients compared to controls. Downregulation in CML patients compared with healthy controls. Downregulation in CLL patients compared with healthy controls.	[92]
*BMAL1*		It forms a heterodimer with CLOCK and NPAS2. This heterodimer binds to E-box enhancing elements upstream of the Period (PER1, PER2, PER3) and Cryptochrome (CRY1, CRY2) genes and activates the transcription of these genes. PER and CRY proteins heterodimerize and repress their own transcription by interacting in a feedback loop with CLOCK/BMAL1 or NPAS2/BMAL1 complexes.	Downregulation in newly diagnosed patients with AML and with relapse of the disease, compared to controls. Downregulation in newly diagnosed and end of treatment from ALL patients compared to controls. Downregulation in newly diagnosed CML patients compared with healthy controls. Downregulation in CLL patients compared with healthy controls.	
*CRY1*		This gene encodes a flavin adenine dinucleotide-binding protein that is a key component of the circadian core oscillator complex, which regulates the circadian clock	Downregulation in patients upon completion of treatment for AML and with relapse of the disease, compared to controls. Downregulation in newly diagnosed and end of treatment from ALL patients compared to controls. Downregulation in newly diagnosed CML patients compared with healthy controls. Downregulation in CLL patients compared with healthy controls.	
*CRY2*			Downregulation in newly diagnosed AML patients and patients upon completion of treatment for AML, and upregulation in patients with relapse of the disease, compared to controls. Downregulation in newly diagnosed and end of treatment from ALL patients compared to controls. Upregulation in CML patients upon 3-months course of chemotherapy, compared with healthy controls.	
*CLOCK*		The protein encoded by this gene plays a central role in the regulation of circadian rhythms. The protein encodes a transcription factor of the basic helix-loop-helix (bHLH) family and contains DNA binding histone acetyltransferase activity. The encoded protein forms a heterodimer with BMAL1 that binds E-box enhancer elements upstream of Period (PER1, PER2, PER3) and Cryptochrome (CRY1, CRY2) genes and activates transcription of these genes	Downregulation in patients with AML, compared to controls. Downregulation in patients newly diagnosed with ALL compared to controls. Downregulation in newly diagnosed CML patients compared with healthy controls. Downregulation in CLL patients at the end of treatment, compared with healthy controls.	
*REV-ERB* *α*		This gene encodes a transcription factor that is a member of the nuclear receptor subfamily 1. The encoded protein is a ligand-sensitive transcription factor that negatively regulates the expression of core clock proteins. This protein represses BMAL1. This protein may also be involved in regulating genes that function in metabolic, inflammatory, and cardiovascular processes.	Downregulation in patients with AML compared with healthy samples. Downregulation in newly diagnosed and end of treatment from ALL patients compared to controls. Downregulation in newly diagnosed CML patients compared with healthy controls. Downregulation in CLL patients compared with healthy controls.	
*PPAR* *α*		PPARs affect the expression of target genes involved in cell proliferation, cell differentiation, and immune and inflammation responses.	Downregulation in newly diagnosed and end treatment from AML patients compared to samples from healthy individuals. Downregulation in newly diagnosed and end of treatment from ALL patients compared to controls. Downregulation in newly diagnosed CML patients compared with healthy controls.	
*CRY-1*	PB and BM samples were collected from 100 CLL patients	This gene encodes a flavin adenine dinucleotide-binding protein that is a key component of the circadian core oscillator complex, which regulates the circadian clock	40 CLL patients showed up-regulation of CRY-1 54 CLL patients showed down-regulation of CRY-1 6 CLL patients had undetectable CRY-1 expression	[93]
*SHARP1* (BHLHE41 or DEC2)	ML-2 cell line derived from a patient with AML; and 14 cases of MLL-AF6 and 42 cases of other subtypes of MLL-rearranged AMLs	This gene is a basic helix-loop-helix transcription factor that acts as a transcription repressor of clock genes and clock-controlled genes.	Up-regulated in MLL-AF6 AML patients compared to cases of other subtypes of AML and cases of normal BM CD34+ cells	[94]
*NPAS2*	Two AML cell lines (MV4-11 and MOLM-14); and human hematopoietic cells from 34 AML patients and 16 healthy controls	This gene is a transcription factor that encodes a protein which will heterodimer with BMAL1 and form core circadian clock genes.	Upregulated in both AML cell lines and in AML patients, compared to controls	[95]
*PER2*	Neutrophils isolated from the peripheral blood samples collected from 30 CML patients and 30 healthy donors	Encodes components of the circadian rhythms of locomotor activity, metabolism, and behavior.	Downregulated in patients with CML compared with healthy individuals	[96]

ALL: Acute Lymphocytic Leukemia; AML: Acute Myeloid Leukemia; BM: Bone Marrow; CLL: Chronic Lymphocytic Leukemia; CML: Chronic Myeloid Leukemia; PB: Peripheral Blood.
